# Tetraspanin CD9 alters cellular trafficking and endocytosis of tetraspanin CD63, affecting CD63 packaging into small extracellular vesicles

**DOI:** 10.1016/j.jbc.2025.108255

**Published:** 2025-02-03

**Authors:** Leanne C. Duke, Allaura S. Cone, Li Sun, Dirk P. Dittmer, David G. Meckes, Robert J. Tomko

**Affiliations:** 1 Department of Biomedical Sciences, Florida State University College of Medicine, Tallahassee, Florida, USA; 2 Department of Microbiology and Immunology, The University of North Carolina at Chapel Hill, Chapel Hill, North Carolina, USA; 3 EV Biomedical, Tallahassee, Florida, USA

**Keywords:** CD9, CD63, exosomes, extracellular vesicles, tetraspanin, tetraspanin-enriched microdomains, trafficking

## Abstract

Small extracellular vesicles (sEVs) are particles secreted from cells that play vital roles both in normal physiology and in human disease. sEVs are highly enriched in tetraspanin proteins, such as CD9 and CD63, and contain tetraspanin-enriched membrane microdomains involved in loading of sEVs with macromolecule cargoes and in sEV biogenesis. However, the precise roles of individual tetraspanins in sEV biogenesis and cargo loading remain poorly understood. Here, we report that CD9 negatively regulated CD63 trafficking to tetraspanin-enriched microdomains and its subsequent packaging into sEVs, whereas CD63 had no discernable effect on CD9 localization or packaging. Using super resolution microscopy of individual vesicles, we showed that CD9 governs the fraction of sEVs that are loaded with CD63. Interestingly, CD9-dependent suppression of CD63 packaging was rescued by pharmacological blockade of endocytosis. Together, our data support a model where CD9 contributes to the regulation and secretion of CD63 in an endocytosis-dependent manner to reprogram the contents of sEVs and tetraspanin-enriched microdomains.

Extracellular vesicles (EVs) are membrane-enclosed particles secreted from cells that regulate basic biological processes such as cell communication, signaling, and transfer of functional cargo from cell to cell (1, 2, 3). EVs have garnered substantial interest because of their potential as vehicles for efficient delivery of molecules to recipient cells and because they have been implicated in the pathophysiology of several human diseases, including cancer, neurodegenerative disorders, autoimmune disorders, and viral infection (1, 3, 4, 5, 6, 7, 8, 9). Thus, mechanisms controlling EV biogenesis and packaging of their cargo are of substantial medical interest.

EVs can be produced by several mechanisms and can vary tremendously in size and contents (1, 2, 10), making attempts to define and differentiate EV populations based on size, mechanism of biogenesis, or contents challenging (10). At present, no universal methods for distinguishing EV populations have been identified. Despite this, three distinct groups of EVs are generally recognized. The first population, known as microvesicles or ectosomes, is formed by budding and pinching off from the plasma membrane (3, 9). These vesicles vary in size from ∼100 to 1000 nm in diameter (9, 10, 11). The second population, called apoptotic bodies, is generally greater than 2000 nm in diameter and is released as membrane blebs from apoptotic cells (9, 11). A final population, called exosomes, typically ranges from ∼50 to 150 nm in diameter and is formed by the inward budding of the late endosome/multivesicular body (MVB). This is followed by fusion of the MVB with the plasma membrane to release the intraluminal vesicles into the extracellular space (3, 8, 12). Despite their different membranes of origin, distinguishing exosomes from smaller microvesicles has been challenging because of their overlap in size and macromolecule content. Further, they rely on similar cellular machinery for biogenesis which has been extensively reviewed (1, 3, 9, 11). Due to the difficulty in distinguishing small microvesicles from exosomes, we will refer to these vesicles jointly as small extracellular vesicles (sEVs).

Biogenesis of sEVs can occur through pathways independent of the endosomal sorting complex required for transport (ESCRT). Such ESCRT-independent sEV biogenesis is typically stimulated by membrane-embedded tetraspanin proteins such as CD9 and CD63, or *via* the actions of specific lipids, such as ceramides (13, 14, 15, 16, 17). Ceramides are thought to induce membrane curvature that promotes spontaneous membrane budding (3, 15, 16). Interestingly, although much larger than ceramides, tetraspanins likely also induce localized regions of high membrane curvature by forming lateral multimeric complexes within the membrane (18, 19, 20). Indeed, tetraspanins are often found enriched in sEVs and the cellular membranes harboring these multimers, called tetraspanin-enriched microdomains (TEMs) (21, 22, 23). TEMs have been shown to serve as sites for the release of sEVs into the extracellular space (14, 21, 24). Consistent with these observations, tetraspanins CD9, CD63, and CD81 are commonly used as markers of sEVs (1, 14, 25).

Although it is clear that tetraspanins are enriched in sEVs and at sites of sEV egress from the cell membrane, the relative contributions of individual tetraspanins to sEV biogenesis and the factor(s) governing their abundances in sEVs remain poorly understood. In this work, we investigated the role of one tetraspanin, CD9, in the biogenesis and trafficking of protein cargoes to sEVs and TEMs. CD9 has vast functionality including in membrane fusion (26, 27, 28), motility (29, 30), proliferation (31, 32), adhesion (33, 34), signaling (35, 36), differentiation (37, 38), trafficking (39, 40), viral infection (41, 42), and invasion (43, 44). Some of these functions have been directly associated with CD9’s incorporation into TEMs or its ability to regulate interactions or activity within the domain, possibly by inclusion or exclusion of molecules in TEMs (45, 46, 47, 48, 49, 50). CD9 can interact with many different proteins or molecules within TEMs including integrins, metalloproteinases, immunoglobulin-superfamily receptors, and other tetraspanins (32, 34, 49, 50, 51). The amount of possible interactions with CD9, which can be direct or indirect, make CD9 distinct from most tetraspanins (45, 49). Its high enrichment in TEMs and sEVs also supports the importance of its incorporation into these domains and its subsequent packaging into sEVs. Therefore, further understanding of the role of CD9 provides insight into sEV packaging and trafficking and also supports a role of TEMs and their relevance to the vast functionality of CD9.

We find that knockdown or overexpression of CD9 reciprocally impacted packaging of CD63 into sEVs *via* TEMs. In contrast, modulation of CD63 levels did not enhance packaging of CD9 into sEVs, suggesting a specific role for CD9 in CD63 packaging rather than a general compensatory mechanism among tetraspanins. Interestingly, the decrease of CD63 packaging into sEVs could be rescued by pharmacological inhibition of endocytosis. This suggests endocytosis of CD63 is not required for its incorporation into sEVs and limits its secretion. Together, these data support a model in which CD9 overexpression promotes, while knockdown restricts, the endocytosis of CD63 which alters its trafficking and incorporation into sEVs.

## Results

### Modulation of tetraspanin CD9 expression inversely affects tetraspanin CD63 cellular levels

**Figure 1:**
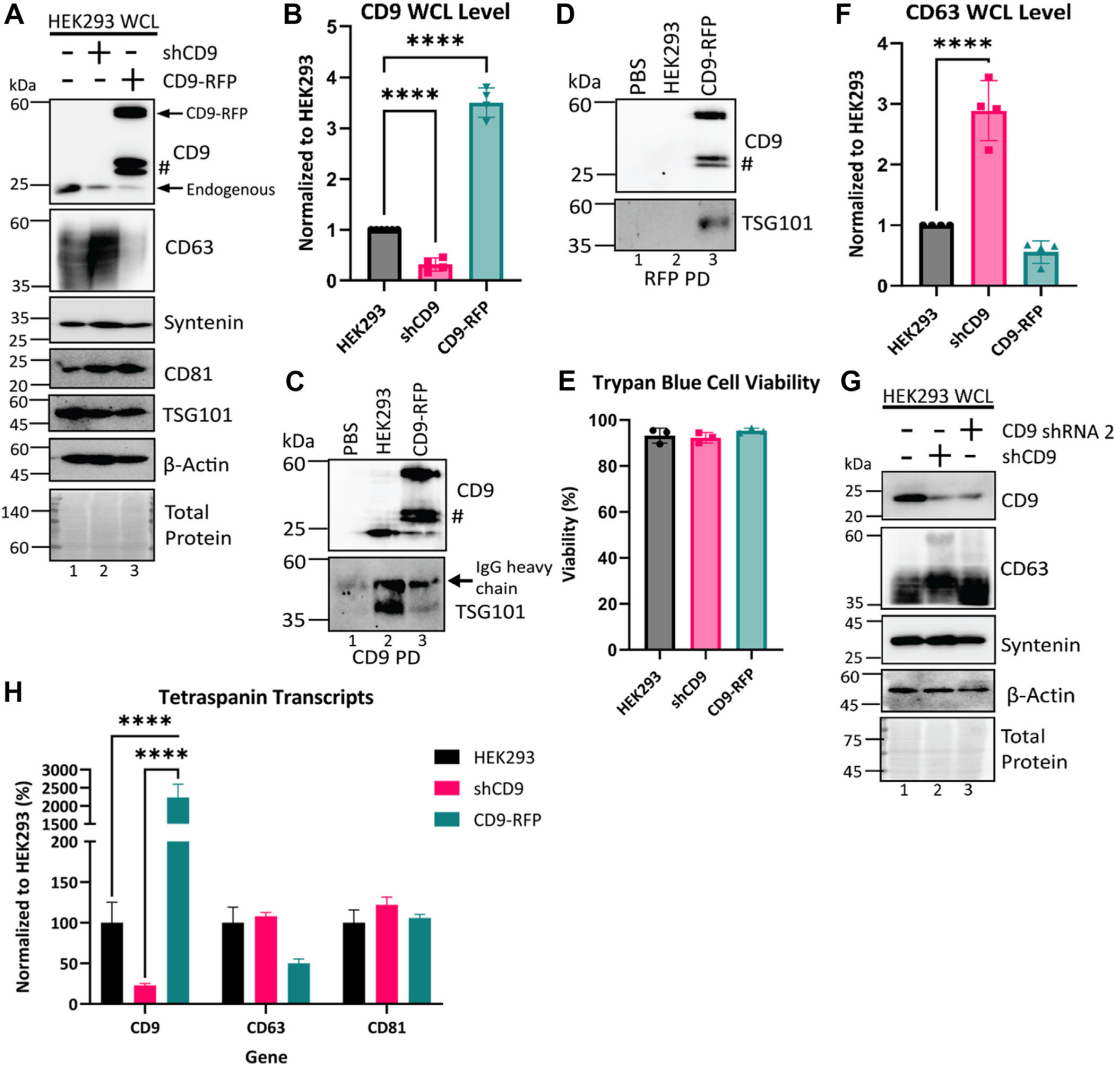
**Knockdown or overexpression of CD9 inversely affects CD63 cellular expression.***A*, representative images of the Western blot analysis of whole-cell lysates (WCLs) from HEK293 cells expressing a CD9 shRNA (shCD9) or RFP-tagged CD9 (CD9-RFP). #, truncation products of CD9-RFP. *B*, quantification of WCL protein levels of CD9 from immunoblot analysis normalized to HEK293 WT (*N* = 4, biological replicates). Statistical significance was determined by one-way ANOVA with a *post hoc* Tukey’s multiple comparisons test; ∗∗∗∗, *p* < 0.0001. *C* and *D*, WCL from HEK293 WT and CD9-RFP were immunoprecipitated using (*C*) anti-CD9–coated Dynabeads or (*D*) RFP-Trap magnetic agarose beads before immunoblotting with the indicated antibodies. The *arrow* indicates IgG heavy chain from the CD9 antibody. (*N* = 2, biological replicates). *E*, the cell viability of each cell line was measured by trypan *blue* staining. *Error bars* indicate SD (*N* = 3, biological replicates). *F*, quantification of WCL CD63 protein levels relative to HEK293 WT (*N* = 4, biological replicates). Statistical significance was determined by one-way ANOVA with a *post hoc* Tukey’s multiple comparisons test; ∗∗∗∗, *p* < 0.0001. *G*, Western blot analysis of WCL from HEK293 cells expressing shCD9 or transfected with a second CD9-targeted shRNA. (*N* = 2, biological replicates). *H*, cellular mRNA transcripts of tetraspanins commonly enriched in sEVs, CD9, CD63, and CD81 were measured by quantitative polymerase chain reaction (qPCR). Values were normalized to HEK293 WT and analyzed by one-way ANOVA with a *post hoc* Tukey’s multiple comparisons test. *Error bars* indicate the SD (*N* = 3, biological replicates); ∗∗∗∗, *p* < 0.0001. sEV, small extracellular vesicle.

To examine the role of tetraspanin CD9 on protein packaging into sEVs, two complementary genetic approaches were taken. First, a doxycycline-inducible CD9-targeted shRNA (shCD9) construct was transduced into HEK293 cells. We confirmed that introduction of doxycycline reduced CD9 protein levels (Fig. 1*A* lane 2, *B*). In the second approach, we overexpressed CD9 with a C-terminal RFP tag in HEK293 cells (CD9-RFP). Although this construct has been validated previously (52, 53), we performed two immunoprecipitation experiments to ensure that CD9-RFP retained interaction with known binding partners. Using magnetic beads coated in anti-human CD9 antibodies that bound both endogenous and exogenous forms of CD9, we successfully coimmunoprecipitated (co-IP) known CD9 binding partner TSG101 from HEK293 and CD9-RFP cells (Fig. 1*C* lane 2 and 3). To ensure that TSG101 specifically interacted with the CD9-RFP fusion, RFP-trap magnetic beads were used to isolate only CD9-RFP. The RFP co-IP showed that CD9 successfully pulled down TSG101 from CD9-RFP lysates (Fig. 1*D* lane 3), but not from HEK293 lysates or a PBS control (Fig. 1*D* lane 1 and 2). In agreement with previous work (52, 53), we concluded the RFP tag did not obviously impair function of CD9. Additionally, neither CD9-RFP expression nor endogenous CD9 knockdown affected cell viability as determined by trypan blue exclusion (Fig. 1*E*).

We next investigated how knockdown and overexpression of CD9 impacted known sEV cargo proteins in whole-cell lysates (WCLs). Immunoblot analysis of WCL showed a decrease in tetraspanin CD63 upon CD9 overexpression (Fig. 1*A* lane 3, *F*). Reciprocally, CD63 was significantly increased in the WCL of CD9 knockdown cells (Fig. 1*A* lane 2, *F*). The increase in CD63 observed in WCL was also observed in Western blots of cells expressing a second CD9 shRNA targeting a different region of the CD9 coding sequence, suggesting this effect was due to CD9 suppression rather than off-target effects (Fig. 1*G*). This effect appeared to be selective for CD63, as the ESCRT pathway protein TSG101 and the adaptor subunit protein syntenin failed to show this reciprocal relationship. Similarly, we observed no impacts of CD9 modulation on another tetraspanin protein, CD81, or on the cytoskeletal protein beta-actin (Fig. 1*A*).

We considered that the changes in CD63 protein levels may derive from altered transcription. To test this, we measured mRNA transcripts of CD9, CD63, and CD81 upon CD9 modulation (Fig. 1*H*). As expected, shCD9 cells showed greatly reduced CD9 mRNA levels and CD9-RFP had elevated transcript levels. In contrast, both CD63 and CD81 transcripts changed less than two-fold in shCD9 or CD9-RFP cells, suggesting the changes observed in CD63 protein levels were primarily posttranscriptional (Fig. 1, *A* and *H*).

### CD9 alters CD63 packaging into sEVs

**Figure 2:**
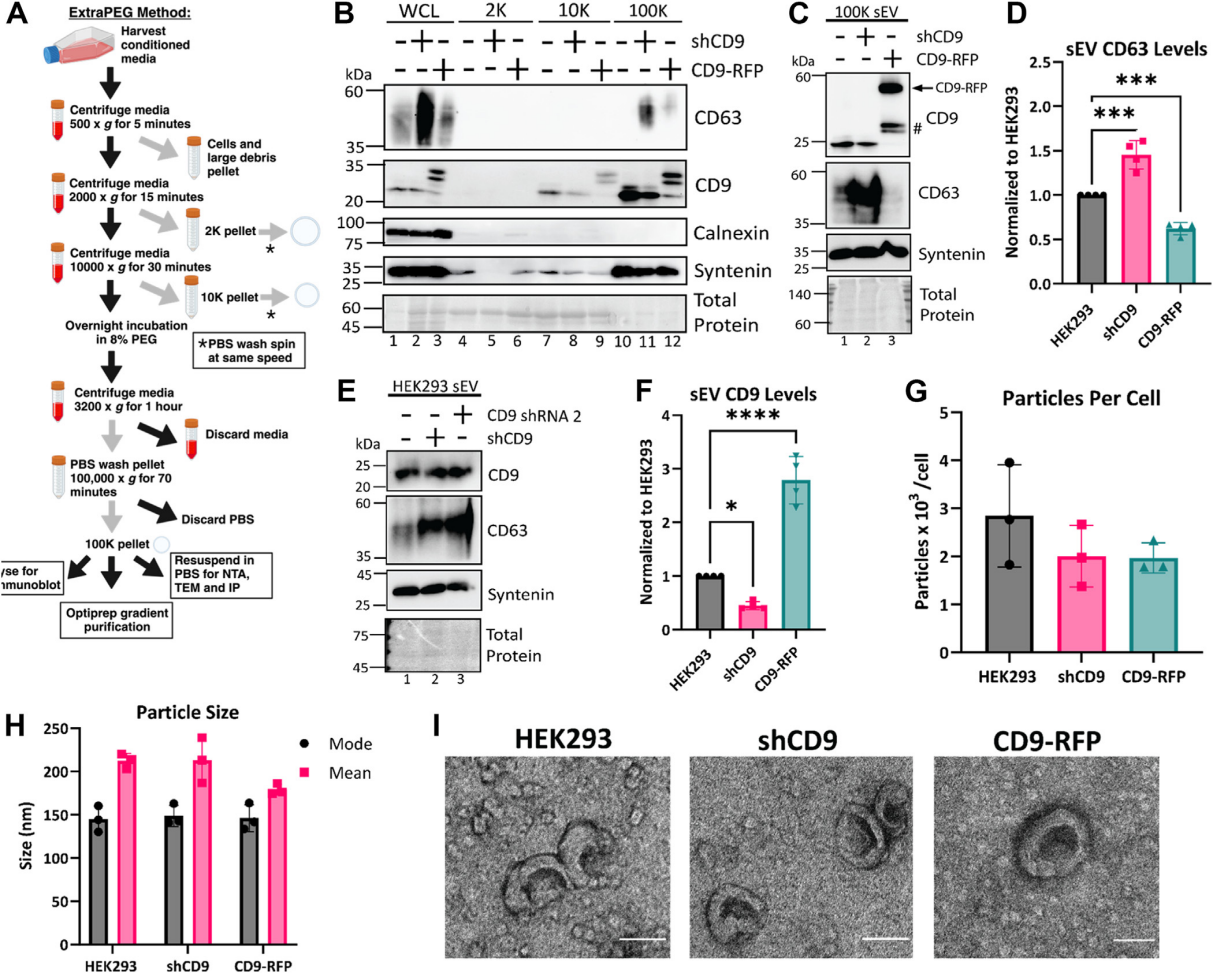
**CD9 alters CD63 packaging into small extracellular vesicles.***A*, BioRender diagram of sEV purification using the ExtraPEG method, highlighting the collection of different EV subpopulations: 2K pellet, which is enriched in apoptotic bodies; 10K pellet, which is enriched in microvesicles; and the 100K pellet, which is enriched in small extracellular vesicles. All pellets were washed with PBS prior to analysis. *B*, Western blot analysis of the different EV populations derived from HEK293 WT, shCD9, or CD9-RFP. Calnexin represents a negative marker for sEVs. (*N* = 2, biological replicates). *C*, Western blot analysis of 100K sEV pellets from HEK293 WT, shCD9, and CD9-RFP. (*N* = 4, biological replicates). *D*, Western blot quantification of CD63 in sEVs, normalized to HEK293 WT (*N* = 4, biological replicates). Statistical significance was determined by one-way ANOVA with a *post hoc* Tukey’s multiple comparisons test; ∗, *p* < 0.05; ∗∗∗∗, *p* < 0.0001. *E*, Western blot analysis of sEVs from HEK293 cells expressing shCD9 or transfected with a second CD9-targeted shRNA. (*N* = 2, biological replicates). *F*, Western blot quantification of CD9 in sEVs, normalized to HEK293 WT (*N* = 4, biological replicates). Statistical significance was determined by one-way ANOVA with a *post hoc* Tukey’s multiple comparisons test; ∗, *p* < 0.05; ∗∗∗∗, *p* < 0.0001. *G* and *H*, the size and concentration of sEVs were quantified by nanoparticle tracking analysis from three biological replicates measured with three technical replicates. Data are represented as particles harvested per cell (*G*) or mode and mean (*H*). Cell counts from trypan exclusion were used to calculate particles harvested per cell. *I*, transmission electron micrographs of the 100K pellet from HEK293 cells stably expressing an inducible CD9 shRNA or CD9-RFP as shown. Images were taken at 40,000x magnification. Scale bars represent 100 nm. (*N* = 2, biological replicates). EV, extracellular vesicle; sEV, small extracellular vesicle; shCD9, CD9-targeted shRNA.

Both CD9 and CD63 are known to be enriched in sEVs (1, 3, 14). We thus considered that CD9 may be influencing the packaging of CD63 into sEVs or other EVs. To test this, we isolated and characterized different populations of EVs produced from shCD9 and CD9-RFP cells using the ExtraPEG method (detailed in Fig. 2*A*) (54, 55, 56, 57). This method combines differential centrifugation and a polyethylene glycol concentration step to enrich EV subtypes from conditioned media. After initial centrifugation steps and PEG precipitation, a 100,000*g* wash step is performed to remove protein contaminants. In this approach, sequential 2,000*g*, 10,000*g*, and 100,000*g* centrifugations pellet different vesicle subpopulations. These 2K, 10K, and 100K pellets are enriched for apoptotic bodies, larger microvesicles, and sEVs, respectively. The contents of EVs derived from each cell type were then assessed using immunoblotting.

To first establish whether the protein contents of a particular EV subpopulation were altered by changes to CD9 expression, we performed immunoblot analysis on proteins from the purified EV subpopulations and compared them to an equal amount of WCL protein (Fig. 2*B*). Calnexin, an endoplasmic reticulum transmembrane protein known to be excluded from EVs (2), was enriched in the WCL, but was absent from the 2K, 10K, and 100K fractions as anticipated (Fig. 2*B*). This indicates the purity of the EV enrichment procedure. As observed previously, CD63 was found primarily in the sEV (100K) fraction, with little to no detectable CD63 in the 2K or 10K fractions (Fig. 2*B* lane 4–12). However, the abundance of CD63 in the 100K fraction was substantially enriched by CD9 knockdown (Fig. 2*B* lane 11, *C* lane 2, *D*), whereas syntenin, another protein enriched in sEVs, was largely unaffected (Fig. 2, *B* and *C*). Inversely, CD9 overexpression significantly decreased CD63 packaging into the 100K fraction (Fig. 2*B* lane 12, *C* lane 3, *D*). This suggests that CD9 selectively but negatively regulates the packaging of CD63 into sEVs. In agreement, a similar enhancement of CD63 in the 100K fraction was observed in cells expressing the second CD9 shRNA (Fig. 2*E*).

Interestingly, although CD9 levels were substantially decreased in the WCL of shCD9 cells, the overall abundance of CD9 in sEVs was only ∼45% lower than in the WT HEK293 sEVs (Fig. 2, *B*, *C* and *F*) after normalizing for total protein content. This was also observed in sEVs from cells expressing the second CD9 shRNA (Fig. 2*E*). However, CD9 was decreased in the 10K fraction of shCD9 and was undetectable in any of the 2K fractions (Fig. 2*B*). This suggests that when CD9 is decreased, the small amount available is preferentially packaged into sEVs rather than other EV types.

Notably, CD9 overexpression or suppression had no appreciable impact on the size and abundance of sEVs in the culture media, as inferred from nanoparticle tracking analysis (Fig. 2, *G* and *H*). Additionally, transmission electron microscopy of sEVs from the 100K pellet showed the cup-like morphology resulting from sample dehydration that is typically associated with sEVs (Fig. 2*I*) (2), but no overt differences in their morphologies. Together, these data indicate that CD9 levels within sEVs are tightly regulated, but CD9 levels have no observed effect on secretion, size or morphology of sEVs.

### CD9 expression in cells and sEVs is unaffected by depletion of CD63

**Figure 3:**
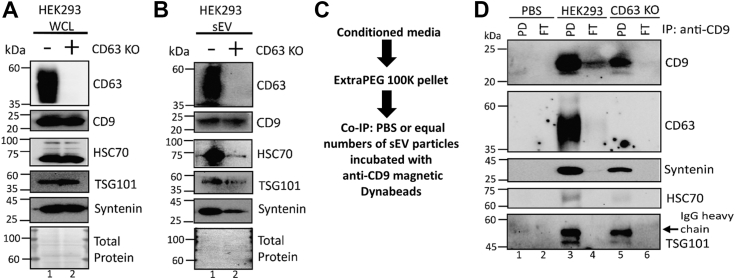
**CD9 levels in cells and small extracellular vesicles are not enhanced by depletion of CD63.***A* and *B*, immunoblot analyses of (*A*) WCL from HEK293 WT and HEK293 CD63 CRISPR (CD63 KO) cells or (*B*) sEVs to assess CD9 protein enrichment. (*N* = 2, biological replicates). *C*, schematic for coimmunoprecipitation (co-IP) of CD9-containing sEVs from HEK293 WT or CD63 KO cells. PBS or an equal number of sEVs were incubated with anti-CD9 magnetic Dynabeads overnight at 4 °C. PBS alone served as a control to distinguish any nonspecific binding. Samples were then placed on a magnetic rack for separation and washes. The washes and supernatant left after the samples were placed on the rack to separate the CD9^+^ vesicles were spun at 100,000*g* and lysed to run as the flow-through (FT). *D*, the pull-down (PD) and FT fractions were analyzed by Western blot with the indicated antibodies. (*N* = 2, biological replicates). Oversaturation of the beads likely caused CD9 to appear in the FT fraction of HEK293 WT. The *arrow* indicates the anti-CD9 IgG heavy chain above TSG101 band. sEV, small extracellular vesicle; WCL, whole-cell lysate.

Previous studies have suggested tetraspanins can functionally compensate for each other, often *via* increased expression of one tetraspanin when another is depleted (34, 58, 59, 60). Our results thus far are consistent with such a possibility. Therefore, we next investigated if altering CD63 expression reciprocally impacted CD9 levels. To assess whether CD63 impacted CD9 expression or packaging into sEVs, we used a previously described (8, 61) HEK293 cell line in which CD63 had been disrupted using CRISPR/Cas9-mediated gene editing (CD63 KO). The abundance of CD9 in the WCL and sEV was not noticeably affected by CD63 KO, whereas other sEV markers TSG101, syntenin, and HSC70 were decreased in sEVs (Fig. 3, *A* and *B*). These markers have been previously shown to be affected by knockout of CD63 (61). Since knockout of CD63 can affect total particle secretion (8, 61, 62), we utilized nanoparticle tracking analysis and co-IP to isolate CD9^+^ vesicles from an equal number of particles from HEK293 WT and CD63 KO cells. Anti-CD9–coated beads were used to isolate the sEVs containing CD9 from WT and CD63 KO purified sEVs (100K), as diagrammed in Figure 3*C*. As expected, CD9 was detected in sEVs from WT and CD63 KO cells but was absent in the PBS control (Fig. 3*D* lane 1–5). Some minimal CD9 was detected in the flow-through of HEK293 WT, likely because the binding capacity of the beads was exceeded. The CD9^+^ sEVs also contained CD63, syntenin, HSC70, and TSG101, indicating that CD9 and CD63 are found in the same sEV subpopulation. Together, these data suggest that loss of CD63 does not enhance CD9 expression and packaging to sEVs, thus this relationship is not reciprocal or compensatory. Instead, this implies that CD63 packaging is dependent on CD9.

### CD9 alters the apparent density of sEVs

**Figure 4:**
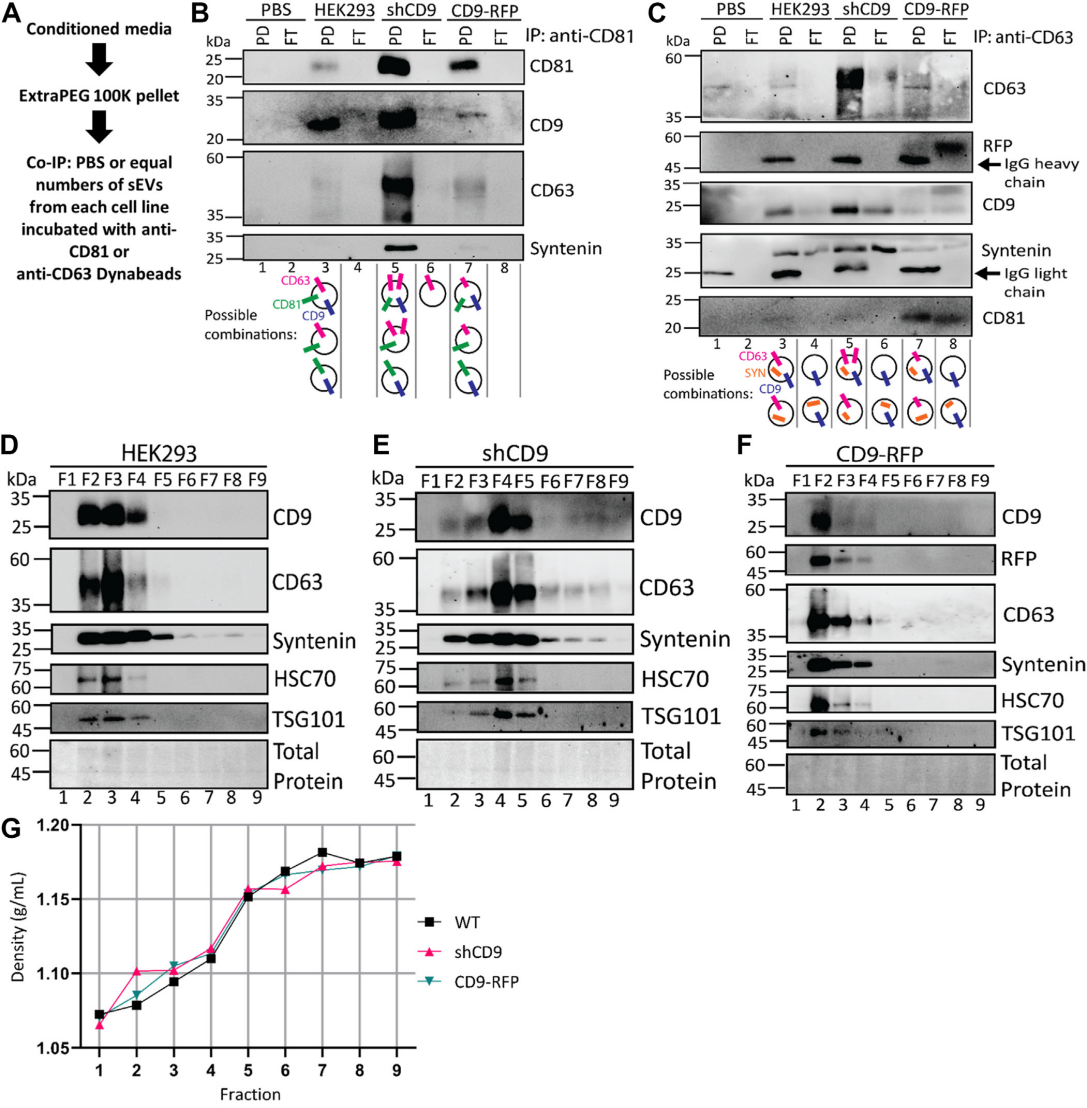
**CD9 alters migration of small extracellular vesicle markers and enrichment of CD63 in small extracellular vesicles.***A*, diagram of co-IP of CD81-containing or CD63-containing sEVs. The 100K pellets from HEK293 WT, shCD9, or CD9-RFP cells were resuspended in PBS and particles were counted by nanoparticle tracking analysis. PBS or an equal number of sEVs from each cell line were then incubated with anti-CD81 magnetic Dynabeads or anti-CD63 magnetic Dynabeads overnight at 4 °C. The next day, PD and FT were collected. The PD and FT fractions of the co-IP with (*B*) CD81 or (*C*) CD63 were then analyzed by Western blotting as indicated. (*N* = 3, biological replicates). Possible combinations of the indicated proteins in sEV populations suggested by the blots are shown. *Arrows* indicate IgG heavy or light chains from the antibody-coated beads. *D*–*F*, Western blot analyses of density gradient purified sEVs from (*D*) HEK293 WT, (*E*) shCD9, or (*F*) CD9-RFP cells. The 100K ExtraPEG pellets from each cell line were fractionated on iodixanol gradients to separate particles by density. Immunoblots of the indicated fractions are shown. Fractions were extracted from each gradient at equal volumes and separated as shown. (*N* = 3, biological replicates). *G*, the densities of each fraction from the gradients shown in (*D*–*F*) were measured by refractometry and plotted. Similar densities were observed for fractions from each gradient separation. Co-IP, coimmunoprecipitation; FT, flow-through; PD, pulldown; sEV, small extracellular vesicle; shCD9, CD9-targeted shRNA.

We continued to utilize co-IP to distinguish different sEV subpopulations based on their tetraspanin profiles. We first purified sEVs by ExtraPEG as described above. Immunoblot analysis showed little alteration to tetraspanin CD81 due to changes in CD9 expression, therefore we first examined CD81 vesicles (Fig. 1*A*). From the purified sEV population (100K), we immunopurified CD81^+^ sEVs using an anti-CD81 affinity resin as diagrammed in Figure 4*A*. Nanoparticle tracking was used to determine equal number of particles from each cell line. Finally, we probed lysates of the CD81^+^ sEVs for the presence of tetraspanins CD63 or CD9 to establish whether they were present in CD81^+^ sEVs. As shown in Figure 4*B*, the CD81^+^ sEVs also contained CD9, CD63, and the sEV marker protein syntenin. Although these experiments did not directly address whether CD81, CD9, and CD63 can coexist in a single sEV, they did demonstrate that CD81^+^ sEVs also contain CD9 and/or CD63. Importantly, the abundance of CD63 in CD81^+^ sEVs from shCD9 cells was increased as observed above (Fig. 4*B* lane 5). CD63 levels in CD9-RFP were similar to WT; this varies somewhat from what we have observed previously. However, this could result from differences in abundances of CD81^+^ vesicles from WT or CD9-RFP cells.

Additionally, this was supported by co-IP performed to pull down CD63^+^ vesicles (Fig. 4*C* lane 7 and 8), where higher CD81 levels were observed in CD9-RFP sEVs containing CD63. Pulldown of CD63^+^ vesicles was performed with anti-CD63 affinity resin, and we similarly found the presence of CD81, CD9, and syntenin in these CD63^+^ sEVs (Fig. 4*C*). As observed with the CD81^+^ sEVs, CD63 was most enriched in sEVs from cells expressing the CD9 shRNA. Interestingly, the flow-throughs from the CD63 purifications, which contained sEVs presumably devoid of CD63, were also reactive with antibodies to CD81, CD9, and syntenin (Fig. 4*C* lane 4, 6, and 8). This suggested that these tetraspanins can be packaged into sEVs lacking CD63. Thus, multiple populations of sEVs containing different combinations of tetraspanins can form, and the relative enrichment of particular tetraspanins correlates with CD9 expression levels. Evidence of tetraspanins cobudding in various combinations in the same sEVs has been validated by various methods (63, 64, 65). From this analysis, we suggest possible combinations of sEV subpopulations (Fig. 4, *B* and *C* cartoons); however, it should be acknowledged that these combinations only account for CD9, CD63, CD81, and syntenin, likely drastically underestimating the true diversity of sEV populations.

We next evaluated the density of sEVs using an iodixanol gradient as previously described (66). In this gradient, sEVs typically migrate to the top, which correlates to lower density fractions (*e.g.*, fractions 2 and 3 in Fig. 4, *D*–*F*). Equal volumes were taken for each fraction. Analysis of the sEVs from WT cells showed sEV marker proteins comigrated in fractions 2 through 4 as shown previously by others (61, 66, 67, 68), with CD63 being more enriched in fractions 2 and 3 (Fig. 4*D* lane 2 and 3). Fractions from CD9-RFP and shCD9 sEVs also contained sEV markers. However, the migration of sEV proteins in the gradient was altered. The sEVs from the shCD9 gradient were more diffuse, with higher enrichment of markers in fractions 4 and 5 (Fig. 4*E* lane 4 and 5). CD63 was present in fractions 2 through 8, but was the highest in fractions 4 and 5, similar to the other markers (Fig. 4*E*). Conversely, CD9-RFP sEVs migrated to fractions 2 through 4, similarly to WT, but were more enriched in fraction 2 compared to fraction 3 for WT (Fig. 4*D* lane 3, *F* lane 2). This was not due to differences in the density gradient, as density measurements of each fraction were consistent among the different separations (Fig. 4*G*). The shift in enrichment of sEV markers thus suggests a change in the density of sEVs themselves. Taken together, these observations indicate that the composition and density of sEVs changes with CD9 levels and that CD63 abundance generally tracked with other known sEV markers.

To directly quantify the sEVs that contain CD63 and CD9, we used quantitative EV surface marker profiling by 2D direct stochastic optical reconstruction microscopy (dSTORM), super resolution microscopy. The exceptional resolution of this approach allows for imaging of single particles (69, 70, 71, 72). To visualize sEVs, we used Cell Mask Deep Red (CMDR) to stain the sEV membrane, distinguishing sEV particles from any protein aggregates, and anti-CD63 antibodies conjugated to Alexa Fluor 488 to visualize CD63. Colocalization of CMDR and green thus indicates the presence of CD63 in a given single sEV. For sEVs derived from CD9-RFP cells, the red fluorescence from RFP was activated using the 561 laser, whereas the CMDR was activated by the infrared 640 laser. Thus, we could simultaneously measure CD9-RFP and CD63 in a given single sEV. The presence of bright green or red fluorescence colocalized with CMDR, within a 200 nm diameter and that could be clearly distinguished from neighboring sEVs were counted. Due to orientation of the particles, colocalization could directly overlap or be separated due to the 10 nm size of the antibodies used for CD63 labeling, as well as the mobility of the RFP fusion to CD9 afforded by a flexible linker sequence. At least 1500 sEVs from each cell line were imaged and analyzed from three separate frames, allowing for percentages of the total sEVs containing CD63 or CD9 to be determined.

**Figure 5:**
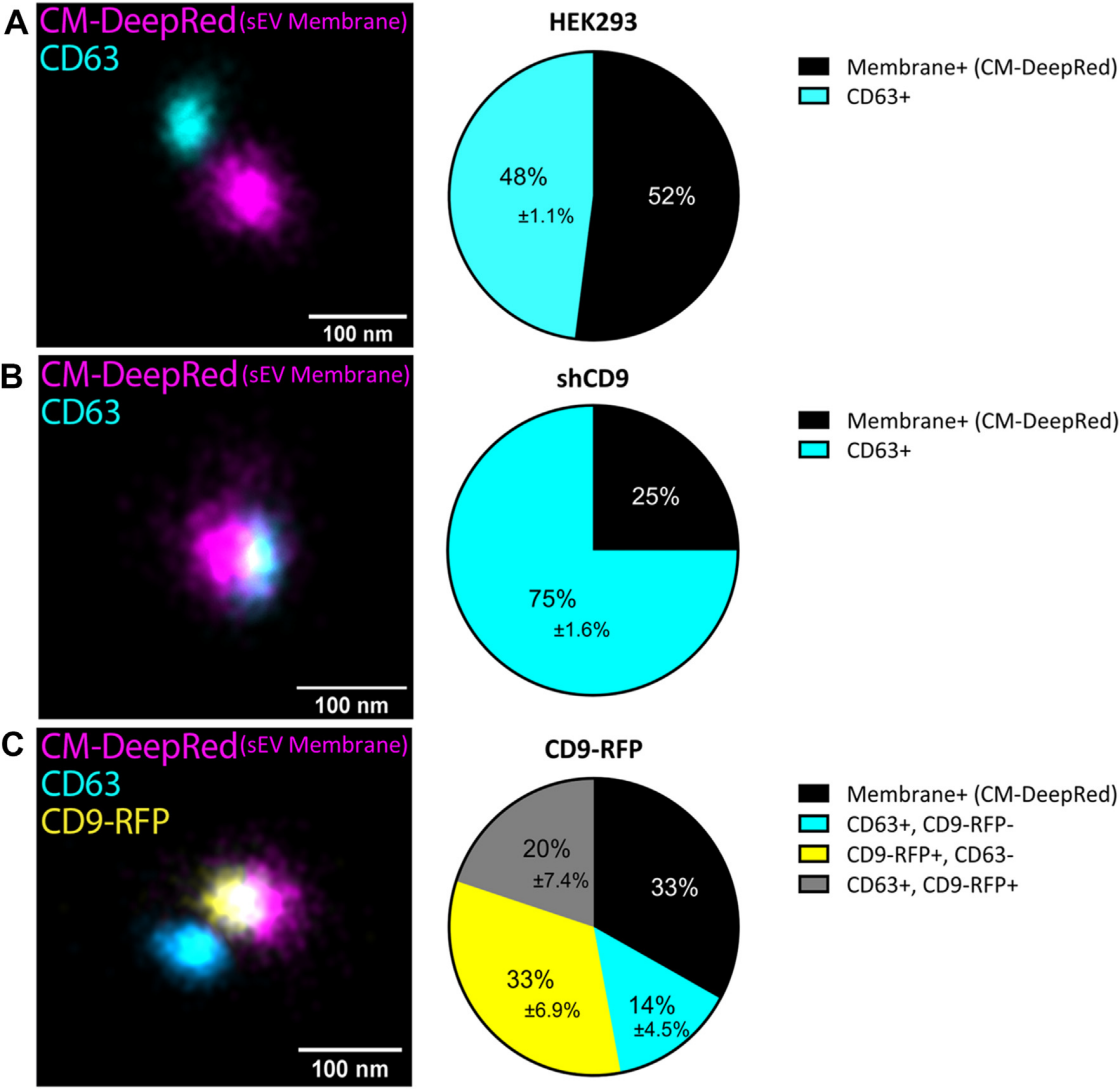
**dSTORM analysis of CD63 in small extracellular vesicle particles.***A*–*C*, quantitative sEV surface marker profiling by 2D direct stochastic optical reconstruction microscopy (dSTORM) super resolution microscopy. Representative dSTORM images of (*A*) HEK293 WT, (*B*) shCD9, or (*C*) CD9-RFP sEV samples labeled with the membrane dye CM-*Deep Red* (CMDR, *magenta*), anti-CD63 antibodies (*cyan*), or CD9-RFP (RFP fluorescence; *yellow*). The 100K pellet of each cell line was seeded onto Ibidi eight-well glass-bottom chamber slides. The sEVs were stained with CMDR and a CD63 antibody that was preconjugated with Alexa Fluor 488 secondary antibody. After staining, the sEVs were washed and placed in oxygen scavenging buffer (B3; ONI) and imaged using the Nanoimager from Oxford Nanoimaging. For particles to be counted bright *green* or *red* fluorescence was colocalized with CMDR within a 200 nm diameter. Some separation in the colocalization of CD63 fluorescence was allowed due to the antibody size. Percentages of membrane+ and CD63+ sEVs in HEK293 WT and shCD9; membrane+, CD63+, CD9+, and double-positive sEVs in CD9-RFP samples were quantified by CODI analysis (ONI Inc.). Membrane+ represents particles only stained by CMDR, therefore representing sEVs presumably devoid or a signal below detection of CD63 or CD9. Experiment performed on one sample, measuring over 1500 sEV particles from three separate fields of view. Pie charts include percentages for each population and percent error representing the SEM. Scale bars represent 100 nm. CMDR, Cell Mask Deep Red; sEV, small extracellular vesicle; shCD9, CD9-targeted shRNA.

In WT sEVs, CD63 was found in 48% ± 1.1% of sEVs stained with CMDR (Fig. 5*A*). When sEVs derived from shCD9 cells were analyzed, the fraction of sEVs that were CD63^+^ increased to 75% ± 1.6% (Fig. 5*B*), consistent with our biochemical analyses. Conversely, the percentage of sEVs derived from CD9-RFP cells that were CD63^+^ decreased to 34% total. Of that total, 14% ± 4.5% of those sEVs contained only CD63 and 20% ± 7.4% contained both CD63 and CD9. Of the remaining 66% that lacked CD63, 33% ± 6.9% contained CD9 and 33% were seemingly devoid of both tetraspanins, instead being only CMDR-positive (Fig. 5*C*). This indicates that there is a population of sEVs that does not contain either tetraspanin. However, it is possible some of these sEVs that appear only CMDR-positive contain some tetraspanins that were not detected due to a signal below detection limits or because they were not marked with their cognate fluorescent antibodies. To ensure a real signal from background, filters were set on the images taken using the CODI software (https://alto.codi.bio/user/login) as described previously (70). This threshold can especially affect detection of the RFP tag on CD9, therefore possibly underestimating CD9 counts in CD9-RFP. While we were unable to readily quantify CD9^+^ CD63^+^ double-positive sEVs isolated from WT and shCD9 cells, we can confidently conclude that modulation of CD9 alters the fraction of sEVs that are CD63^+^. Together, these data demonstrate at single-particle resolution that CD63 packaging into sEVs is reciprocally related to CD9 expression.

### CD9 alters trafficking of CD63 to sEVs and TEMs

**Figure 6:**
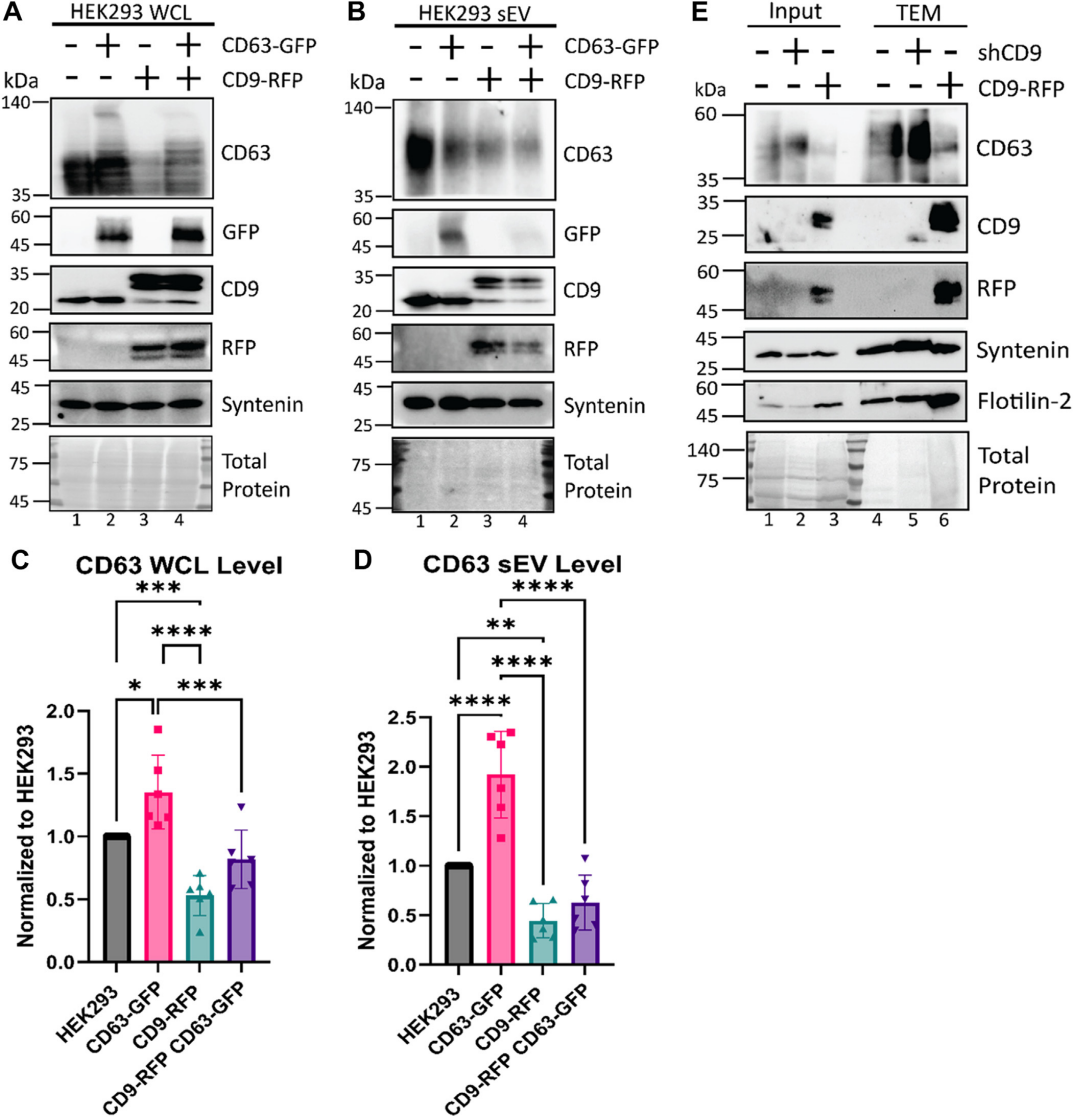
**CD9 alters trafficking of CD63 to small extracellular vesicles and tetraspanin-enriched microdomains.***A* and *B*, Western blot analysis of (*A*) WCL or (*B*) sEVs from HEK293 WT and CD9-RFP cells expressing CD63-GFP or not. Cells were transfected with CD63-GFP to restore loss of CD63 cellular expression in CD9-RFP cells. *C* and *D*, quantification of CD63 levels from (*C*) WCL blots in (*A*) above or (*D*) sEV protein levels from (*B*) above. CD63 levels in each sample were normalized to HEK293 WT (*N* = 6, biological replicates). Statistical significance was determined by two-way ANOVA with a *post hoc* Tukey’s multiple comparisons test; ∗, *p* < 0.05; ∗∗∗∗, *p* < 0.0001. *E*, immunoblot analysis of tetraspanin-enriched microdomains (TEMs) isolated from HEK293 WT, shCD9, and CD9-RFP cells by sucrose gradient centrifugation. Cells from three 150 mm plates were suspended in Brij-97, homogenized, and loaded on the bottom of the gradient in 40% sucrose/MNE buffer. Thirty percent of sucrose/MNE was then carefully layered, followed by 5% sucrose/MNE. After centrifugation, the top 2 ml was discarded, saving the next 2 ml fractions to be washed and then lysed. For input, WCL from the starting material for the sucrose gradient were loaded at equal protein. (*N* = 3, biological replicates). sEV, small extracellular vesicle; shCD9, CD9-targeted shRNA; WCL, whole-cell lysate.

To further validate that CD9 is altering sEV packaging of CD63, we investigated the effect of overexpressing CD63 as a C-terminal GFP fusion (CD63-GFP) on its packaging. We reasoned that if the significantly decreased incorporation of CD63 into sEVs in CD9-RFP cells was solely due to the lower cell expression levels of CD63, then overexpression of CD63-GFP in the cell would be anticipated to restore CD63 packaging in CD9-RFP sEVs. As expected, overexpression of CD63-GFP significantly increased total CD63 levels in WT cells (Fig. 6*A* lane 1 and 2, *C*). While immunoblots showed CD63 levels did increase within the WCL of CD9-RFP, overexpression did not fully rescue expression to WT levels (Fig. 6*A* lane 3 and 4, *C*). Similarly, CD63 packaging into sEVs was not recovered (Fig. 6, *B* and *D*). While there is some variation in quantitation due to different transfection efficiency of the CD63-GFP construct, we can conclude CD63 packaging remained deficient (Fig. 6, *B* and *D*). This further supports the hypothesis that CD9 modulates CD63 sEV packaging, and the packaging deficit is not solely due to the decrease of cellular CD63 available to be packaged.

To deduce how CD9 may be modulating the packaging of CD63 into sEVs, we examined the expression and subcellular localization of CD9 and CD63 *via* confocal microscopy. CD9 is known to localize mainly to the plasma membrane, whereas CD63 is found primarily in the endolysosomal system (25, 73). However, it should be noted that through membrane recycling and fusion with vesicles, CD63 can be found on the plasma membrane and CD9 on the endosomal membrane, albeit typically in lower abundances. Comparison of micrographs from WT, shCD9, and CD9-RFP cells indicated no CD9-dependent change to endogenous or overexpressed CD63 localization (Fig. S1, *A* and *B*). Both proteins exhibited normal localization; however, as expected CD9 has enhanced or low expression due to the overexpression or knockdown, respectively. Overexpression of CD9 enhanced its enrichment on the plasma membrane and in compartments inside the cells in comparison to WT (Fig. S1).

One additional possibility is that CD9 changes the movement and trafficking of CD63 such that it no longer traffics effectively to sEV packaging sites. We focused on TEMs, which are compartmentalized areas on the membrane enriched in tetraspanins and other select proteins. TEMs are known to support vesicle biogenesis, protein sorting, and vesicle loading (14, 21, 24). As CD9 and CD63 are abundant in both TEMs and sEVs, we examined if trafficking of CD63 to TEMs was altered by modulation of CD9 levels. TEMs were isolated from cells by manual lysis in Brij 97 detergent, and then using sucrose gradient centrifugation followed by a PBS wash spin to pellet material (74, 75, 76). Although this gradient has been used in previous studies (77, 78), it should be noted that manual disruption could allow for contaminating proteins to copurify with TEMs. However, CD9 and CD63 have been identified in multiple studies to be contained in TEMs (14, 21, 23), so any effect on our assay is likely minimal. TEMs isolated from CD9-RFP cells showed a decrease in CD63 enrichment compared to WT TEMs but showed an expected increase in CD9 enrichment (Fig. 6*E*). Conversely, shCD9 TEMs had increased CD63 expression. However, no changes were observed in the abundances of syntenin or the membrane microdomain scaffolding protein flotilin-2, commonly enriched in TEMs, compared to total protein levels (Fig. 6*E*). Therefore, these data suggest that CD9 alters trafficking of CD63 to TEMs, which in turn likely modulates CD63 incorporation into sEVs.

### Altered trafficking of CD63 to sEVs is recovered by pharmacological disruption of endocytosis

To further investigate alteration to trafficking, we utilized chemical inhibitors of various vesicular trafficking steps. Interestingly, when cells were treated with chloroquine, which inhibits acidification of the lysosome to block endolysosomal fusion, we were able to partially recover WCL levels of CD63 in CD9-RFP, but not its packaging into sEVs (Fig. S2). This is consistent with results above; recovery of cellular CD63 does not fully restore secretion. Rab7, a GTPase that controls transport of materials to the lysosome, was used to verify inhibition of fusion. Secretion of Rab7 was increased by chloroquine treatment and brightfield images show increased vesiculation due to inhibition of fusion of endosomes with the lysosome (Fig. S2). These data suggest that CD63 is being trafficked and degraded in the lysosome, decreasing WCL levels in cells overexpressing CD9, but this is not the cause of the sEV packaging defect.

Recent studies showed that endocytosis limits the secretion of several exosome marker proteins, including CD81, CD9, and CD63 (79, 80). These studies suggested that vesicular secretion of these tetraspanins occurred mainly from the plasma membrane, as secretion of CD63 was strongly induced by inhibitors of endocytosis. Whether this was mediated through CD9 was not tested. Therefore, we investigated whether disrupting endocytosis would recover CD63 sEV packaging in CD9-RFP cells. We utilized three mechanistically distinct pharmacological inhibitors to disrupt endocytosis: latrunculin A (Lat A), an actin polymerization inhibitor; dynasore, a dynamin inhibitor; and chlorpromazine, a compound that has been reported to inhibit the AP-2 adaptor complex required for clathrin-mediated endocytosis (79, 80, 81). Notably, the C terminus of CD63 is reported to interact with several AP complexes, including μ2 of AP-2 (82).

**Figure 7:**
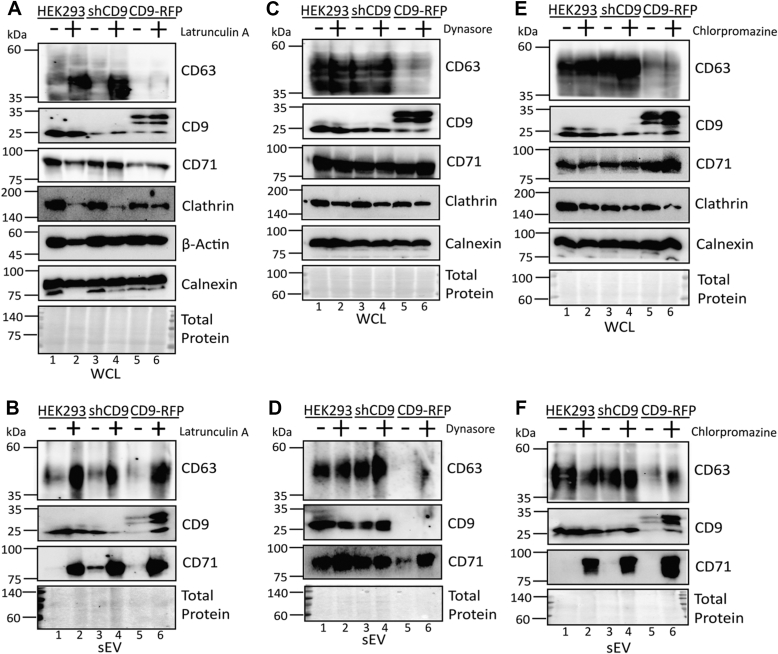
**Altered trafficking of CD63 to small extracellular vesicles in CD9-RFP is recovered by pharmacological disruption of endocytosis.***A* and *B*, Western blot analysis of (*A*) WCL and (*B*) sEVs from HEK293 WT, shCD9, and CD9-RFP cells treated with 1 μM actin polymerization inhibitor latrunculin A (Lat A) for 24 h prior to analysis. Calnexin, an ER-resident protein, is shown as a control. (*N* = 4, biological replicates). *C* and *D*, Western blot analysis of (*C*) WCL or (*D*) sEVs from HEK293 WT, shCD9, and CD9-RFP cells treated with 30 μM dynamin inhibitor dynasore for 24 h prior to analysis. (*N* = 4, biological replicates). *E* and *F*, Western blot analysis of (*E*) WCL and (*F*) sEVs from HEK293 WT, shCD9, and CD9-RFP cells treated with 10 μM chlorpromazine, an inhibitor of AP-2–dependent clathrin-mediated endocytosis, for 24 h prior to analysis. (*N* = 4, biological replicates). sEV, small extracellular vesicle; WCL, whole-cell lysate.

The resident ER protein calnexin, which does not enter the secretory pathway, was unaffected in WCL by Lat A treatment (Fig. 7*A*). We used the transferrin receptor CD71 as a marker for endocytosis. As anticipated, CD71 was enriched in sEVs from all cell lines after treatment with Lat A (Fig. 7*B*). Importantly, treatment with Lat A increased CD63 in both the WCL and sEVs of all cell lines (Fig. 7, *A* and *B*), suggesting a role for endocytosis in regulation of CD63 levels. Treatment with dynasore had a modest, if any, effect on CD63 in CD9-RFP WCL, but had no effect on CD63 expression in WT or shCD9 WCL (Fig. 7*C*). Additionally, dynasore recovered CD63 sEV packaging in CD9-RFP and further enhanced CD63 secretion in shCD9 (Fig. 7*D*). Lastly, chlorpromazine completely recovered CD63 packaging in CD9-RFP sEVs, potentially at the expense of cellular CD63 levels (Fig. 7, *E* and *F*). CD71 levels in sEVs were enhanced by chlorpromazine treatment in every cell line (Fig. 7*F*). Whereas all three inhibitors restored CD63 sEV secretion similar to WT levels, they were not all able to fully recover cellular expression in CD9-RFP cells. This is consistent with our previous results (Fig. 6, *A*–*D*) showing restoration of WCL expression of CD63 does not recover the packaging defect. These data further support the notion that CD9 modulates the trafficking of CD63 into sEVs rather than the lack of secretion being caused solely by decreased cellular CD63. Taken together, blocking endocytosis of CD63 restores its secretion despite CD9 overexpression, presumably be preventing its routing for lysosomal destruction and instead directing it to TEMs.

## Discussion

Our data show that CD9 modulates the trafficking of CD63 to TEMs and thereby regulates its packaging into sEVs. Although changes to trafficking of CD63 into TEMs have been previously reported, our findings provide a previously unappreciated mechanistic link between tetraspanins in sEV packaging and secretion. Interestingly, both CD9 and CD63 are known to form complexes in TEMs with other proteins like integrins that aid in signaling and other functions. Specifically, studies established that CD63 translocated from granules to the plasma membrane to form a complex with CD9-α_IIb_β_3_ in TEMs on the surface of activated platelets and this complex modulated platelet spreading on fibrinogen (83, 84). This study has shown a similar phenomenon where CD63 is trafficked to TEMs at different levels by changes in CD9 expression, which then promoted CD63 secretion (Fig. 6*E*). However, our work suggests that CD9 serves an antagonistic role for delivery of CD63 to TEMs; thus, cell type–specific mechanisms may exist to control CD63 localization and packaging into sEVs.

Additionally, our evidence reinforces the hypothesis that TEMs are important sites for sEV biogenesis and protein trafficking into sEVs (14, 21, 24) as the abundance of CD63 in sEVs correlated with its expression in the TEMs (Figs. 2 and 6*E*). Our findings that packaging of CD63 was not recovered upon CD63 overexpression (Fig. 6, *A*–*D*) or plasma membrane enrichment *via* blockade of endocytosis (Fig. 7) suggests that the trafficking of CD63 to certain domains on the membrane is more critical than its overall expression level. Although we found a correlation between expression of CD63 in TEMs and its level of secretion, it is important to note this study did not directly assess whether TEMs were fully responsible for the biogenesis of CD63-containing sEVs. However, our data suggest that they are likely a major site in some cell types, given the high enrichment of cellular CD63 in TEMs in our experiments. Further characterization of the roles of TEMs and individual tetraspanins in sEV biogenesis will be necessary to address this possibility.

To fully understand TEMs, it will be necessary to address sources of experimental variation, such as the use of different detergents for cell lysis prior to sucrose gradient purification. Certain detergents will preserve some complexes while disrupting others, making identification of all TEM components by large scale methods, such as proteomics, more challenging (85, 86). Additionally, transient interactions are likely to be missed or excluded. This exemplifies the need to further verify interactions by different methods, such as co-IP, which has been commonly used in studies of TEMs. However, to fully understand the role of TEMs and the individual tetraspanins composing them, new methods need to be established to study individual TEMs within the cell.

Alterations to CD9 did not affect the size or morphology of the sEVs but did affect the sEV populations containing syntenin and tetraspanins CD9, CD63, and CD81 produced from HEK293 cells (Figs. 2 and 4). An observed shift in the migration of sEV markers by iodixanol gradient further confirmed density changes to the sEV populations induced by CD9 modulation (Fig. 4, *D*–*G*). The changes in the density and cargo in sEVs induced by CD9 modulation indicates CD9 likely has a larger role in regulation of sEV biogenesis than currently appreciated. CD63 has been of high interest in studies of exosomes and other sEVs due to its high enrichment in endosomes, which are believed to be a major source of secreted sEVs, compared to CD9 and CD81, which localize more to the plasma membrane and are generally associated with small microvesicles. CD63 is involved in exosome biogenesis and trafficking of proteins throughout the endolysosomal system as well as to exosomes (61, 62, 73, 80). Additionally, it was recently shown CD63 has a role in sorting cholesterol to endosomes for storage or distribution through exosomes (87). Therefore, tight regulation of CD63 is likely very important for its role in vesicle biogenesis, but also in the trafficking and regulation of other proteins and molecules. Furthermore, CD9 is known to modulate the trafficking of other proteins into sEVs (45, 88, 89). Therefore, it is likely that CD9’s effect on CD63 trafficking contributes to the regulation and secretion of CD63 and its possible interaction partners, resulting in changes to the sEV populations as we have observed here.

To assess whether the fraction of sEVs loaded with CD63 was altered by CD9, we profiled sEV contents by dSTORM super resolution microscopy. This revealed that modulation of CD63 in sEVs was caused at least in part by a change in the fraction of CD63^+^ sEVs (Fig. 5). These data further support a shift in the biogenesis of certain sEV subpopulations, rather than solely a change in the amount of CD63 loaded into sEVs. Although our data do not unequivocally address whether individual CD63^+^ sEVs contain altered abundances of CD63 in response to modulation of CD9, we note that the total number, size, and abundance of exosomes was unchanged under these conditions. Thus, the changes in CD63 in sEVs derives either solely from changes to the fraction of sEVs containing CD63, or a combination of changes to the fraction containing CD63 and the number of CD63 molecules present (on average) in sEVs. A quantitative analysis of CD63 abundance in individual sEVs will be necessary to distinguish between these possibilities.

Additionally, our dSTORM data suggest the existence of at least four different sEV populations, as characterized based on tetraspanin content. This was consistent with our co-IP experiments (Fig. 4, *B* and *C*), but allowed further distinction of subpopulations to compensate for limitations of co-IP. The subpopulations distinguished by dSTORM consisted of CD9^+^ CD63^-^, CD9^-^ CD63^+^, or populations positive or negative for both tetraspanins (Fig. 5). Previous studies using single-particle interferometric reflectance imaging and quantitative single-molecule localization microscopy have supported these observations (63, 64). Given the typical subcellular localizations of CD9 and CD63, we can infer these various subpopulations occur from recycling of proteins through the endolysosomal system back to the plasma membrane where vesicles bud in a stochastic fashion. Our results support secretion of sEVs occuring primarily at the plasma membrane, and for CD63 its packaging is limited by endocytosis. It has generally been thought that CD63 is endocytosed and incorporated into intraluminal vesicles that are then trafficked to the lysosome or back to the plasma membrane for secretion. However, recent studies have brought into question this endocytosis-dependent model of secretion for CD63 (79, 80, 90, 91). These studies have established that secretion of CD63 is increased when its internalization is limited by inhibiting endocytosis (79, 91) or mutagenesis to the lysosomal-targeting motif (25), which is also consistent with our results. We were able to recover packaging of CD63 into sEVs from CD9-RFP cells with three mechanistically different inhibitors of endocytosis (Fig. 7), further supporting this model.

Additionally, a recent study showed that syntenin promoted secretion of CD63 by blocking its endocytosis through competition with the AP-2 complex for CD63 (79, 80). Although we did not directly investigate this possibility in our study, we did observe that syntenin was enriched in TEMs with CD63 and CD9. Therefore, TEMs may function as sites to limit or enhance interaction between CD63 and syntenin to control CD63 endocytosis and secretion. We note that these observations are contrary to an exosomal pathway of secretion of CD63, as inhibiting endocytosis would be expected to impair CD63 release rather than enhance it. Further studies will be needed to understand the relationship between CD63, syntenin, the AP-2 complex, and CD9.

Together, this leads us to conclude that secretion of CD63 and likely other vesicular cargo occurs more efficiently at the plasma membrane and is limited by endocytosis. It is reasonable to speculate that secretion of microvesicles, which occurs directly from the plasma membrane, would be faster or more efficient than exosome secretion, which requires formation of intraluminal vesicles in the MVB that subsequently traffic back to the plasma membrane for fusion and release. Secretion of proteins present on the plasma membrane in sEVs would thus be predicted to occur more readily, which is supported here and by other studies (25, 79, 91). Despite this, the contribution of exosomes from the endosomal pathway to tetraspanin secretion is likely to be important, especially under some circumstances. For example, plasma membrane expansion to replace membranes lost from direct secretion may become limiting in some circumstances, resulting in a comparative increase in exosome formation. Further studies will be needed to address these questions, as well as improved methods for profiling the content and abundances of proteins in single sEVs.

## Experimental procedures

### Cell culture

HEK293 (American Type Culture Collection; CRL-1573) and HEK293 CD63KO (8, 61) cells were cultured in Dulbecco’s Modified Eagle Medium (Sigma; D5796). Media were supplemented with 10% fetal bovine serum (Gibco; 26140-079), 2 mM *L*-glutamine (Corning; 25-005-Cl), 100 IU penicillin-streptomycin (Corning; 30-002-CI), and 0.25 μg/ml antibiotic/antimycotic (Corning; 30-004-CI). Cells were confirmed to be free of *mycoplasma* contamination by qPCR testing. Prior to use in cell culture, fetal bovine serum was depleted of any contaminating vesicles by centrifugation at 100,000*g* for 20 h and filtering with a 0.2 μm filter. Cell viability was measured at time of harvest by staining with 0.2% trypan blue (Sigma; T8154) in PBS and counted with an automated cell counter (Cellometer Vision, software version 2.1.6.1; Nexcelom Biosciences). Live-cell counts were used for cell viability percentages and to derive particles per cell by nanoparticle tracking analysis, described later.

### Plasmids and cloning

Generation of the CD9 shRNA and CD63 CRISPR cell lines have been detailed previously (61, 92, 93). The shCD9 was stably expressed in HEK293 cells under the control of a tetracycline-inducible promotor by transduction with lentiviral particles packaged using HEK293-T cells. Puromycin at 2 μg/ml (Acros; 58-58-2) and blasticidin at 10 μg/ml (InvivoGen; ant-bl-1) were used to select for cells containing the CD9 shRNA and the tet repressor, respectively. The pLenti XI shRNA plasmid with target sequences underlined:

CD9_shRNA_fwd, 5′-GATCCCCAAGAAGGACGTACTCGAAACGTGTGCTGTCC GTTTCGAGTACGTCCTTCTTG TTTTTGGAAA-3′

CD9_shRNA_rvs, 5′-AGCTTTTCCAAAAACAAGAAGGACGTACTCGAAACGGA CAGCACAC GTTTCGAGTACGTCCTTCTTG GG-3′ were generated by annealing and ligation of primers for recombination into the pLenti XI shRNA plasmid and transformation. The CRISPR guide oligos were annealed and ligated into the pLentiCRISPRv2 vector (Addgene plasmid # 52961). The plasmid was transformed into Endura *Escherichia coli* (Lucigen) and sequenced to confirm its insertion. Lentivirus made using packaging plasmids pMD2.G, pMDLg/pRRE, and pRSV-Rev was harvested from HEK293-T cells and used for transduction of HEK293 cells. The second shCD9 was purchased from MilliporeSigma MISSION Lentiviral predesigned shRNA (MilliporeSigma; TRCN0000057470). The target sequence provided was: 5′-GCTGTTCGGATTTAACTTCAT-3′. HEK293 cells were transfected with the second shRNA using Lipofectamine 3000 (Invitrogen, L3000015) following the manufacturer’s transfection protocol. Lastly, cells were transduced with pCT-CD9-RFP Cytotracer (SBI; CYTO123-PA-1) lentiviral particles and selected with puromycin to create a stable cell line overexpressing CD9-RFP.

### Immunoblot analysis

WCLs were prepared by scaping cells into ice-cold PBS, followed by centrifugation at 500*g* for 10 min, 4 °C. Cells were lysed by incubation in radio-immunoprecipitation assay (RIPA) buffer with protease inhibitor cocktail (Thermo; 78,429) on ice for 30 min. The cell suspension was vortexed every 5 min and then centrifuged at 20,000*g* for 10 min, 4 °C to pellet insoluble material. The lysate was carefully removed to avoid disturbing the pellet. Lysates of sEV samples were prepared in strong lysis buffer (5% SDS, 10 mM EDTA, 120 mM Tris–HCl [pH 6.8], 8 M urea, and protease inhibitor cocktail). Protein levels of the WCL and sEV lysate were quantified using the Invitrogen EZQ protein quantification kit (Thermo; R33200) following the manufacturer’s protocol. For SDS-PAGE, 25 μg of WCLs were typically loaded per lane, except in Figure 2*B* where 10 μg per lane was loaded to mirror the protein amounts from the EV populations. Typically, 5 to 15 μg of sEV protein was loaded per lane, or in some cases, one-third the total sample volume. Equal volume was used only for the sEV iodixanol gradient and co-IP in Figure 4. A nonreducing Laemmli buffer lacking β-mercaptoethanol (Amresco; 60-24-2) or DTT (Amresco; 3483-12-3) was used for analysis of tetraspanins CD63 and CD9. Western blots were performed as previously described (61, 94). Ponceau S stain (Sigma; P7170-1L) was used to visualize total protein before immunoblotting. Blots were probed with the following primary antibodies: CD9 (Millipore; CBL162), CD63 (Invitrogen; 10628D), CD81 (Genetex; 72476), syntenin (Abcam; ab133267), TSG101 (Santa Cruz; SC-7964), CD71 (Cell Signaling; 13113S), β-actin (Cell Signaling; 3700S), RFP (Rockland; 600-401-379), GFP (Rockland; 600-401-215), Calnexin (Santa Cruz; SC-11397), HSC70 (Santa Cruz; SC-7298), and Rab7 (Cell Signaling; 9367S). Secondary antibodies were horseradish peroxidase-conjugated rabbit anti-mouse IgG (Genetex; 213112) and goat anti-mouse IgG (Genetex; 27171). Blots were imaged using chemiluminescence on a Bio-Rad ChemiDoc imager (170-01401) with Radiance Q ECL (Azure biosystems; AC2101) Western blotting substrate. Images were processed using ImageQuant TL v8.2.0.0 software (https://www.cytivalifesciences.com/en/us/shop/protein-analysis/molecular-imaging-for-proteins/imaging-software/imagequant-tl-analysis-software-p-28619?srsltid=AfmBOopE4A3_96u_0Gi6dw85mLCgE-hLlpMjj5VO_eGX_yPDxTziRjh8) for quantitation and Adobe Photoshop CS6 (v2024) and Adobe Illustrator (v2024) to organize and prepare images for the figures.

### Transfection

HEK293 cells were seeded in 100 mm plates and transfected with 4 μg GFP or pCT-CD63-GFP (SBI, CYTO120-PA-1) plasmid, or with plasmids encoding the second CD9 shRNA using Lipofectamine 3000 (Invitrogen, L3000015). Reagents were diluted in Opti-MEM (Gibco, 31985–070) according to the manufacturer’s protocol. Transfection efficiency was estimated using the percentage of GFP-positive cells. Cells were harvested 24 h after transfection and lysed in RIPA buffer for immunoblotting as above.

### Immunoprecipitation

Co-IP of proteins from HEK293 WT and CD9-RFP cells was performed using CD9-conjugated Dynabeads (Thermo; 10614D) or RFP-trap magnetic beads (Chromotek; rtm-20). Cells were lysed in NP-40 lysis buffer (50 mM Tris-Cl, pH 8.0, 150 mM sodium chloride, 0.5% NP-40, and protease inhibitor cocktail). Protein was quantified using the Pierce 660 nm protein assay (Thermo Fisher Scientific; 22660) and equal protein was used for each sample. The beads were washed and incubated with the cell lysates or with PBS control for 1 h with rotation at 4 °C. After incubation, beads were collected using a magnetic tube rack, washed three times with NP-40 lysis buffer, and transferred to a new tube after the last wash. The beads were then resuspended in 2x Laemmli sample buffer and boiled. The supernatant was then separated by SDS-PAGE for immunoblot analysis.

For experiments using sEVs as the input, the pellets from ExtraPEG purification were resuspended in PBS, and nanoparticle tracking was used to determine the sEV concentration of each sample. Equal particles (1 × 10^10^) from each cell line or PBS-only control were then nutated overnight at 4 °C with magnetic Dynabeads conjugated to CD81 (Thermo; 10616D), CD63 (Thermo; 10606D), or CD9 (Thermo; 10614D). The following day, the beads were collected with the magnetic tube rack and the supernatant was transferred to an ultracentrifuge tube (Beckman; 347287). The beads were washed three times with PBS and then transferred to the ultracentrifuge tube. Washes were combined with previously transferred supernatant from the same sample and centrifuged at 100,000*g* for 70 min at 4 °C. This pellet was resuspended in 2x Laemmli sample buffer and run as the flow-through. RIPA buffer was then added to the beads and incubated on ice for 15 min to lyse the immunoprecipitated sEVs before addition of 2x Laemmli buffer for immunoblotting analysis.

### RNA isolation and quantitative PCR

Total RNA from sEVs was isolated using Trizol reagent (Thermo Fisher Scientific, 15596026) following the manufacturer’s protocol. Purified RNA was dissolved in PCR-grade nuclease free water and quantified by Nanodrop. Up to 1 μg of total RNA was converted into complementary DNA (cDNA) by qScript cDNA SuperMix (QuantBio, 95048) in a 20 μl reaction volume. One microliter of the cDNA was used as template in 15 μl quantitative polymerase chain reaction (qPCR) reactions containing 1X PerfeCTa SYBR Green FastMix (QuantaBio, 95072) and 250 nM final concentration gene-specific primers (PrimerBank). The protocol was 95 °C for 5 min, 40 cycles of 95 °C for 10 s, 60 °C for 10 s, 72 °C for 20 s, followed by a melt curve assay to confirm specific product formation in the reaction. The qPCR was carried out on CFX Connect Real-Time PCR Detection System (Bio-Rad). RNA levels were normalized to GAPDH. Primer sequences:

CD63-F:5′-CAGTGGTCATCATCGCAGTG-3′;

CD63-R: 3′-ATCGAAGCAGTGTGGTTGTTT-5′;

CD9-F: 5′-TTCCTCTTGGTGATATTCGCCA-3′;

CD9-R: 3′-AGTTCAACGCATAGTGGATGG-5′;

CD81-F: 5′-TTCCACGAGACGCTTGACTG-3′;

CD81-R: 3′-CCCGAGGGACACAAATTGTTC-5′

### EV isolation and enrichment

EVs were isolated from conditioned cell media by differential centrifugation and PEG precipitation as described previously (54). All centrifugation steps were performed at 4 °C. A diagram detailing the procedure can be found in Figure 2*A*. After initial centrifugation at 500*g* to remove dead cells and large debris, conditioned cell media was centrifuged at 2000*g* for 15 min and 10,000*g* for 30 min. Media was incubated with 16% PEG 6000 (16% [wt/vol] PEG, 1 M NaCl) at a 1:1 ratio (final PEG concentration = 8%) overnight at 4 °C. The following day, the mixture was centrifuged at 3000*g* for 1 h and the supernatant was decanted. The pellet was washed with 1 ml PBS and recentrifuged at 100,000*g* for 70 min in a TLA.120.2 rotor. The PBS was decanted, and the pellet was resuspended in either PBS, lysis buffer, or 0.25 M sucrose in 10 mM Tris (pH 6.4) buffer depending on the experiment.

### Transmission electron microscopy

For negative staining, sEVs resuspended in PBS were dotted onto parafilm to create a small bead on the surface. Copper-coated grids (400 hex mesh copper; Electron Microscopy Sciences [EMS]; 215-412-8400) were placed on top of the sample for 1 h and then washed three times with PBS, using filter paper to gently blot the PBS from the grid. Grids were then prepared and stained as previously described (92, 95). Specifically, grids were fixed with 2% EM-grade paraformaldehyde (EMS; EM grade; 157-4) for 10 min. The grids were washed three times with PBS and incubated in a 2.5% glutaraldehyde solution (EMS; EM grade; 16120) for another 10 min. Grids were washed three times with ultrafiltered water and stained with 2% uranyl acetate (EMS; 22400-2). Then grids were applied to 0.4% uranyl acetate and 0.13% methylcellulose for 10 min. Finally, grids were flipped sample side up and allowed to harden at room temperature for 24 h. Imaging was performed using a Hitachi HT7800 transmission electron microscope at 120 kV and 40,000x magnification.

### Nanoparticle tracking

Nanoparticle tracking was performed as described previously (8, 61) using the Malvern NanoSight LM10 instrument with a camera level of 13. Videos were processed using the NTA 3.4 software (https://www.malvernpanalytical.com/en/support/product-support/software/nanosight-nta-software-update-v3-4-4) with a detection level of 4. Cell counts measured as described above were used to derive particles per cell.

### Iodixanol density gradient

Following the 100,000*g* centrifugation step of the ExtraPEG method, sEVs were resuspended in 1.5 ml of gradient buffer (0.25 M sucrose, 10 mM Tris-Cl, pH 6.4). They were then separated on an iodixanol (Optiprep) (Sigma; D1556) gradient as described previously to further purify the sEVs (66, 67). Briefly, the sEVs were added to the bottom of a polypropylene (9/16 × 3 1/2 inch; Beckman; 331372) tube and mixed at a 1:1 ratio with 60% iodixanol diluted in gradient buffer for a final concentration of 30%. Next, 20% iodixanol in gradient buffer was carefully layered on top, followed by 10% iodixanol in gradient buffer. The layered mixture was then centrifuged in an MLS-50 rotor for 90 min at 268,000*g*, 4 °C using minimum acceleration and deceleration. Eleven 490 μl fractions were taken from top to the bottom of the resultant gradient. The density of each fraction was measured using a refractometer (Refracto 30PX). Fractions were washed with PBS and recentrifuged at 100,000*g* for 2 h, 4 °C in a SW41 rotor. Pellets were resuspended in PBS and used for further analysis. Fractions 1 to 9 were used for immunoblotting. Fractions 10 and 11 were excluded as they primarily consisted of precipitated material and protein aggregates.

### 2D direct stochastic optical reconstruction microscopy

sEV samples were imaged by dSTORM using the protocol previously described (69, 70, 71, 72). sEVs from each cell line were added to a microslide 8-well glass-bottom chamber (iBidi; 80827) and fixed with 0.5% paraformaldehyde. The CD63 antibody (Abcam; ab59479) was conjugated to Alexa Fluor 488 with a labeling kit (Thermo Fisher Scientific; A20181) according to manufacturer’s instructions. A lipid dye, CMDR was incubated with sEVs overnight at 4 °C to stain the sEV membrane, allowing for detection of particles. The prestained sEVs were then incubated with the conjugated CD63 antibody. Samples were imaged using the Nanoimager (Oxford Nanoimaging) described in (71). Data was analyzed using ONI’s CODI analysis platform: https://alto.codi.bio/user/login. CD63 labeled with Alexa Fluor 488 or CD9-RFP signal was counted only if it was colocalized with CMDR to reduce inflation of sEV counts due to noise.

### Purification of membrane microdomains

Cells were harvested from three 150 mm plates and pooled. TEMs were isolated using Brij-97 detergent (Amresco; J125) and a sucrose gradient as described previously (74, 75, 76). Briefly, cells were resuspended in 1% Brij-97 in MNE buffer (25 mM MES, 150 mM NaCl, 5 mM EDTA, pH 6.5) with protease inhibitor cocktail and manually lysed with a loose dounce homogenizer 35 times. Samples were incubated on ice for 30 min and were then centrifuged at 200*g* for 5 min at 4 °C. Supernatants were collected, setting aside some of the sample for the input, and the remainder was added to the bottom of the 12 ml ultracentrifuge tube. Supernatant was mixed 1:1 with 80% sucrose in MNE buffer to create a final concentration of 40% sucrose. Then 30% sucrose, followed by 5% sucrose, was gently layered dropwise with a needle. After layering of each gradient, they were centrifuged at 250,000*g* for 20 h at 4 °C in a SW41 rotor. Minimum acceleration and deceleration were used. The following day, the TEM fraction was collected by discarding the top 2 ml, keeping the next 2 ml, and transferring this fraction to a new tube for a PBS wash. PBS was added and the sample was centrifuged at 150,000*g* for 1 h at 4 °C. The pellet from this spin was resuspended in lysis buffer as above before analysis by immunoblotting.

### Chemical inhibitor treatments

HEK293 cells were seeded in 100 mm plates and incubated with 1 μM Lat A (Abcam; ab144290), 30 μM dynasore (Selleck Chemical; S8047), or 10 μM chlorpromazine (Selleck Chemical; S5749) dissolved in dimethyl sulfoxide, for 24 h before harvesting for analysis as above. For chloroquine experiments, 50 μM chloroquine (InvivoGen; tlrl-chq) dissolved in PBS was added to the cells for 24 h. Brightfield images were taken using the Invitrogen EVOS FL Digitally Inverted Fluorescence microscope immediately prior to harvest for biochemical analyses.

### Cell microscopy

To examine the subcellular localization of CD63, cells were seeded on coverslips in a six-well plate. After induction of shCD9 for 24 h, cells were fixed with ice-cold methanol. Coverslips were incubated with antibodies against CD63 (Invitrogen; 10628D) and CD9 (Millipore; CBL162) followed by secondary antibodies conjugated to Alexa Fluor 488 and Alexa Fluor 594, respectively. Coverslips were mounted on glass slides and imaged using a Zeiss LSM 880 microscope. Cellular localization of CD63 was further observed by seeding cells into 35 mm glass-bottom dishes. The following day, the cells were transfected with CD63-GFP as above. The following day, Hoechst nuclear stain (5 μg/ml; 62249; Thermo Fisher Scientific) was added for 20 min before washing the cells. Imaging was performed using a Zeiss LSM 880 microscope as previously described (67, 93), and images were processed using Zen 2.1 Black software (https://www.zeiss.com/microscopy/us/products/software/zeiss-zen.html).

## Data analysis and statistics

Statistical analysis was performed using GraphPad Prism 10 (GraphPad Software, https://www.graphpad.com/updates/prism-10-1-1-release-notes) with statistical significance considered at *p* ≤ 0.05. Statistical significance was assessed by one-way ANOVA with a *post hoc* Tukey’s multiple comparisons test. Immunoblot images were quantified with ImageQuant TL v8.2.0.0 software before statistical analysis with Graphpad Prism 10.

## Data availability

All data for this study are contained within the article.

## Supporting information

This article contains supporting information.

## Conflict of interest

The authors declare that they have no conflicts of interest with the contents of this article.
