# Supplementary material for: Tetraspanin CD9 alters cellular trafficking and endocytosis of tetraspanin CD63, affecting CD63 packaging into small extracellular vesicles
Source: J Biol Chem. 2025 Feb 3;301(3):108255. doi: 10.1016/j.jbc.2025.108255 (PMC11919600; doi:10.1016/j.jbc.2025.108255)
Supplement: Supporting information [file mmc1.docx]

**Supporting Information**

**Tetraspanin CD9 alters cellular trafficking and endocytosis of tetraspanin CD63, affecting CD63 packaging into small extracellular vesicles**

Leanne C. Duke^1^, Allaura S. Cone^2^, Li Sun^1^, Dirk P. Dittmer^2^, David G. Meckes^3^, Robert J. Tomko Jr.^1^

^1^ Department of Biomedical Sciences, Florida State University College of Medicine, Tallahassee, FL, 32306, United States of America

^2^ Department of Microbiology and Immunology, The University of North Carolina at Chapel Hill, Chapel Hill, NC, 27599, United States of America

3 3122 Mahan Dr. Ste. 801-239, Tallahassee, FL 32308

**Table of Contents**

Supporting Figure S1. Cellular localization of tetraspanins CD9 and CD63

Supporting Figure S2. Treatment with the lysosomal fusion inhibitor chloroquine did not recover CD63 small extracellular vesicle packaging

**
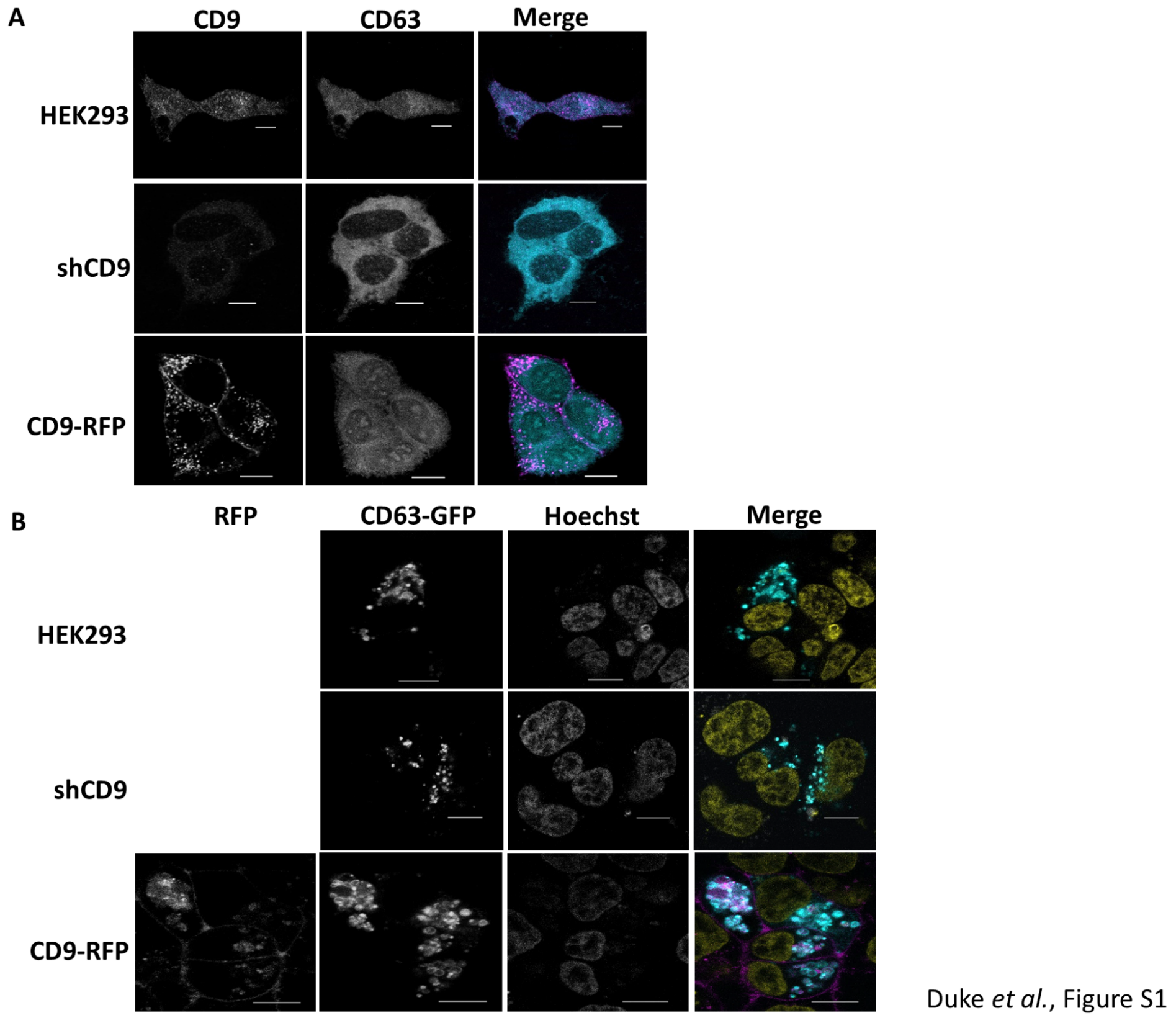
**

**Supporting Figure S1. Cellular localization of tetraspanins CD9 and CD63**

(A), Representative confocal fluorescence images of fixed HEK293, shCD9 or CD9-RFP cells. Cells seeded on coverslips were fixed with cold methanol and stained with CD9 and CD63 antibodies prior to imaging on a Zeiss LSM 880 microscope. Experiment was performed twice. (B), Representative confocal fluorescence images of live HEK293, shCD9 and CD9-RFP cells transfected with CD63-GFP. Cells were seeded into 35 mm glass-bottom dishes and transfected with CD63-GFP. After 24 hours, cells were stained with Hoechst nuclear stain and imaged on a Zeiss LSM 880 microscope. Scale bars = 10 μm. Experiment was repeated once.


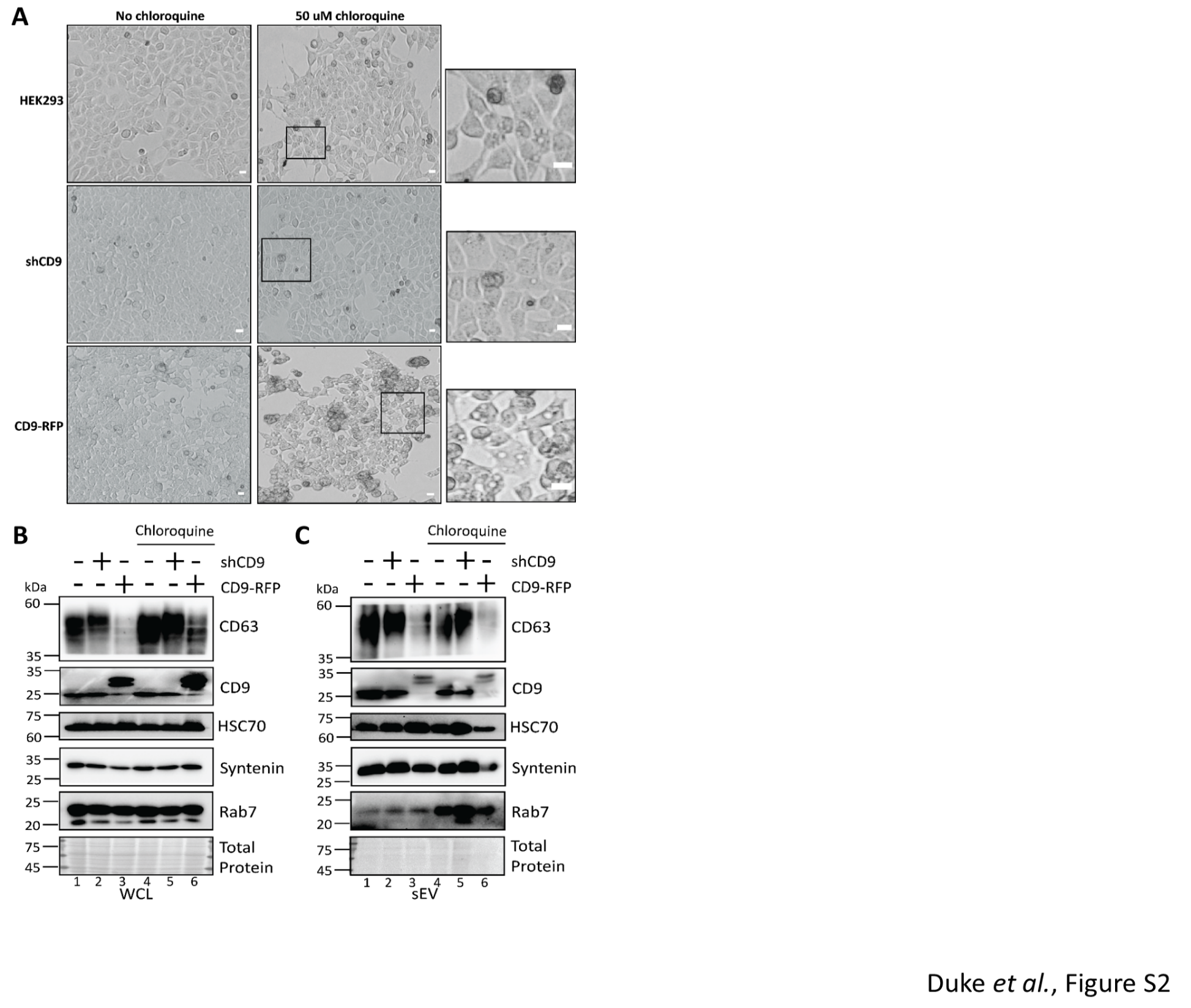


**Supporting Figure S2. Treatment with the lysosomal fusion inhibitor chloroquine did not recover CD63 small extracellular vesicle packaging**

(A), Brightfield images of HEK293, shCD9 or CD9-RFP cells treated with vehicle or 50 μM chloroquine for 24 hours before analysis. Chloroquine inhibits acidification of the lysosome to block endosome-lysosome fusion. Boxes correspond to the magnified images on the right showing increased vesiculation in response to chloroquine treatment. Scale bars = 10 μm. (B, C), (B) WCL or (C) sEVs from HEK293, shCD9 or CD9-RFP cells treated with vehicle or 50 μM chloroquine for 24 hours were subjected to immunoblotting with the indicated antibodies. Immunoblotting was performed on three biological replicates. Rab7 is a marker for late endosomes, which can be shuttled to the lysosome for destruction or to the plasma membrane for fusion. Chloroquine treatment cells prevented Rab7 destruction in the lysosome and increased cell vesiculation due to compromised fusion of late endosomes with the lysosome.
